# Use of Cyclophosphamide, Vincristine, Prednisolone and Vinblastine for the Treatment of Large Cell Lymphoma in Cats

**DOI:** 10.1111/jvim.70066

**Published:** 2025-03-21

**Authors:** Lee Pui Yung Anna, Rodrigo Horta, Cheryl Nathalie Sze, Antonio Giuliano

**Affiliations:** ^1^ Jockey Club College of Veterinary Medicine and Life Sciences City University of Hong Kong Kowloon Hong Kong; ^2^ Department of Veterinary Clinic and Surgery Federal University of Minas Gerais Belo Horizonte Brazil; ^3^ Department of Infectious Diseases and Public Health, Jockey Club of Veterinary Medicine and Life Sciences City University of Hong Kong Kowloon Hong Kong; ^4^ CityU Veterinary Medical Centre Lai Chi Kok Hong Kong; ^5^ Department of Veterinary Clinical Sciences, Jockey Club College of Veterinary Medicine and Life Sciences City University of Hong Kong Kowloon Hong Kong

**Keywords:** cat, chemotherapy, high‐grade, vinca alkaloid

## Abstract

**Background:**

The standard chemotherapy treatment for large‐cell lymphoma in cats is CHOP (cyclophosphamide, doxorubicin, vincristine, and prednisolone) or COP (cyclophosphamide, vincristine, and prednisolone) chemotherapy protocols. Substituting vinblastine for vincristine might have similar efficacy, with lower severity of gastrointestinal adverse events.

**Hypothesis/Objectives:**

To evaluate whether the addition of vinblastine to a low‐dose vincristine COP protocol could reduce the frequency and severity of adverse gastrointestinal effects while maintaining or increasing efficacy.

**Animals:**

Medical records of 41 cats with large‐cell lymphoma treated with the modified COVP protocol at one veterinary referral institution.

**Methods:**

Retrospective case series study. All relevant clinical data were retrospectively collected. Median progression‐free survival, disease‐free interval, and survival time were calculated using the Kaplan–Meier Method. Differences between groups were analyzed using the log‐rank test, and adverse events were graded using VCOG‐CTCAE v2.

**Results:**

Progression‐free survival was 264 days (range, 6–1486 days), the disease‐free interval was 812 days (range, 39–1486 days) and the median survival time for all cats was 412 days (range, 7–1772 days). Complete response was achieved in 59% of the cases, and partial response was observed in 17%. Cats that achieved CR lived significantly longer, 838 days (range, 81–1772 days) versus 143 days (range, 10–798 days; *p* = 0.0018). The COVP protocol was generally well tolerated, and the most common adverse effects were mild signs of gastrointestinal disease and hematological abnormalities that did not require a pause in treatment. Grade‐1 vomiting was the most common (24%), followed by grade‐2 (22%) and grade‐1 reduced appetite (20%).

**Conclusion:**

Cats with lymphoma treated with COVP seem to achieve acceptable survival and response rates compared to traditional chemotherapy protocols.

**Abbreviations**
CHOP: cyclophosphamide doxorubicin vincristine prednisolone
COP: cyclophosphamide, vincristine, and prednisolone
COVP: cyclophosphamide, vincristine, prednisolone, with the addition of vinblastine
CR: complete response
FN: female neutered
MN: male neutered
MST: median survival time
NE: not evaluated
PD: progressive disease
PFS: progression‐free survival
PR: partial response
SD: stable disease
ST: survival time
VBL: vinblastine
VCR: vincristine

## 1 Introduction

Lymphoma is the most prevalent form of cancer in cats, accounting for 30% of all neoplasia and 90% of all hematopoietic neoplasia [1, 2]. With variable anatomic forms, the gastrointestinal tract is the most common site of lymphoma in cats in countries with low prevalence of FeLV [3]. With the rapidly growing and aging group of domestic cats, lymphoma remains an important health concern [4].

The mainstay treatment of most lymphoma in cats is chemotherapy, in which COP (cyclophosphamide, vincristine and prednisolone) and CHOP (cyclophosphamide, vincristine, prednisolone and doxorubicin) chemotherapy protocols are currently the most frequently used [5, 6, 7]. Both COP and CHOP protocols have demonstrated effectiveness, although response rates and survival times might vary. Complete response rates of COP protocols range from 33% to 75%, while CHOP protocols have shown similar complete response rates of 38% to 80% [6, 7, 8, 9, 10]. The duration of remission and survival times achieved with CHOP protocols are comparable to COP protocols. Remission duration with the CHOP protocol is reported to be between 5 to 21 months and survival ranging from 2 to 10 months [6, 7, 8, 9, 10]. Meanwhile, COP protocols reported remission durations of 3 to 8 months and survival times of 2 to 9 months are described [6, 7, 8, 9, 10]. The only study comparing COP and CHOP focused on mediastinal lymphomas and revealed no statistical differences between response rates (62% vs. 67%, respectively) or median survival time (484 vs. 211 days, respectively, *p* = 0.892) [11].

Cats that achieve complete remission usually have longer survival times [6, 7]. However, conventional chemotherapy has failed to make relevant progress in terms of response rate, duration of remission, and survival times [6, 7, 12, 13]. The use of novel chemotherapy protocols is required to improve outcomes and extend survival times. While several previous studies have evaluated the clinical efficacy of incorporating doxorubicin, the addition of vinblastine to the COP protocol as a combination therapy has not been reported.

Vinblastine seems to have similar efficacy in treating lymphoma in cats to vincristine [14]. Despite vincristine and vinblastine having similar mechanisms of action, they are not identical. They often have different indications with different response profiles in different tumors, possibly different mechanisms of resistance, and different toxicity profiles, suggesting that they could have additive or synergistic effects [14, 15, 16]. Results of one study suggest that vinblastine might serve as a viable substitute for vincristine when treating cats with lymphoma, resulting in similar efficacy but fewer gastrointestinal adverse events [14]. However, considering the myelosuppressive effects of vinblastine, the administration of vinblastine in combination with cyclophosphamide, as per COP protocol, could predispose to severe neutropenia. A modified COP protocol (COVP) that uses a lower dosage of vincristine in combination with cyclophosphamide and single‐agent vinblastine could reduce adverse events while maximizing the efficacy of the protocols.

The aim of this study was to assess the safety and clinical efficacy of the COVP protocol.

## 2 Method

### 2.1 Case Selection

The medical record database of CityU Veterinary Medical Centre (VMC) was retrospectively reviewed for cats diagnosed and treated with large‐cell lymphoma presented between 2020 and 2024 (*n* = 41), using the veterinary software RxWorks. Case selection was limited to cats with cytologically or histologically confirmed large‐cell lymphoma treated with the COVP chemotherapy protocol. Cats that met the inclusion criteria and were treated at VMC by COVP for which follow‐up was available for evaluating clinical and objective response were incorporated in this retrospective study.

Animal data extracted from medical records included signalment (age, sex, date of birth, breed, neuter status), presenting clinical signs, weight at presentation, during and after treatment, known medical issues, and laboratory findings, including any abnormalities on CBC or biochemistry and retroviral status using the SNAP Combo Test (antibody for feline leukemia virus and antigen for feline immunodeficiency virus status).

Anatomical location(s) of lymphomas confirmed by cytology or histology were recorded, including any lymph node involvement and/or internal organ involvement, and later further classified into gastrointestinal, nasopharyngeal, renal, mediastinal, liver, larynx, mixed, and multicentric forms. Confirmation was done only at the primary site and not at other sites.

### 2.2 Treatment, Dose and Response

The same protocol was administered to all animals in the study by the same clinician (Table 1). Vinblastine was administered as a single agent, and vincristine (except for the first trial dose) was administered together with cyclophosphamide. Prednisolone was administered at a progressively reduced dosage, and asparaginase was administered before starting the chemotherapy protocol at the discretion of the clinician. The initial intended dose for vincristine was 0.5 mg/m^2^ IV, for vinblastine varied from 1.3 to 1.5 mg/m^2^, and for cyclophosphamide, 220–250 mg/m^2^. The drug dosages were typically increased weekly by approximately 10% of the initial dose, provided no adverse events were observed or reported by the owner, following the standard protocol practiced at the author's hospital. Before every treatment, a complete blood count was performed for all animals. A biochemistry panel, which included renal parameters, was performed for every other cyclophosphamide injection.

**TABLE 1:** COVP protocol and starting doses.

Week	1	2	3	4	5	6	7	8	9	10	11	12	13
Vincristine (0.5–0.6 mg/m^2^ IV)	*	*			*			*			*		*
Vinblastine (1–1.5 mg/m^2^ IV)			*	*		*	*		*	*			
Cyclophosphamide (142–250 mg/m^2^ IV)		*			*			*			*		*
Prednisolone (1.5–2 mg × kg)													

Week	14	15	16	17	18	19	20	21	22	23	24	25	26
Vincristine (0.5–0.6 mg/m^2^ IV)		*		*			*			*			*
Vinblastine (1–1.5 mg/m^2^ IV)													
Cyclophosphamide (142–250 mg/m^2^ IV)		*		*			*			*			*
Prednisolone (1.5–2 mg × kg)													

### 2.3 Response

Clinical response and objective response were evaluated at various points in time. Clinical response was assessed by clinical signs and physical examination, and objective response with different diagnostic imaging modalities, depending on the lymphoma's anatomical form (e.g., renal/gastrointestinal forms using abdominal ultrasonography, mediastinal forms using thoracic radiographies). The assessment was performed weekly at the beginning of the consult; and, if complete response, at the end of the protocol; or at any time if there were any clinical concerns. Objective response was assessed by applying the response characterization criteria established by the Veterinary Cooperative Oncology Group (VCOG) [17]. Complete response (CR) was defined as the absence of all target lesions that were monitored for response to treatment. Partial response (PR) was defined as more than or equal to a 30% reduction in the total diameter of all target lesions. Stable disease (SD) was defined as a reduction in the total diameter of target lesions by less than 30% or an increase in total diameter by less than 20%. Progressive disease (PD) was defined as a 20% increase in the total diameter of all target lesions or the presence of new lesion(s). The CR and PR were categorized as the best point in time, and the total response rate was defined by CR + PR. Adverse events were evaluated based on the grading system from the Veterinary Cooperative Oncology Group—Common Terminology Criteria for Adverse Events (VCOG‐CTCAE v2) [18].

### 2.4 Statistical Analysis

Progression‐free survival (PFS) was defined as the time interval from initiating COVP treatment to either relapse or progression of disease. Disease‐free interval (DFI) was defined as the length from treatment initiation to the last known date of being disease‐free and calculated only in cats with a complete response. Survival time was defined as the length of time from diagnosis to death. Median PFS, DFI, and survival time (MST) were calculated using the Kaplan–Meier product limit method. Cats that were dead from other causes, lost to follow‐up, or remained alive were censored from the PFS, DFI, and survival time analysis. All cats with the intention‐to‐treat that received at least one chemotherapy injection were included.

Comparison of survival data between groups was analyzed using the log‐rank test, and statistical significance was determined by a *p*‐value less than 0.05. GraphPad Prism v. 6.02 was utilized for all statistical analyses.

## 3 Results

In total, 41 cats met the inclusion criteria. The study sample consisted of 21 female cats and 20 male cats. The most represented breeds were Domestic Shorthair (*n* = 24), British Shorthair (*n* = 6), and American Shorthair (*n* = 3); 6 other breeds were also represented (Maine Coon, Scottish Fold, Chinchilla, Ragamuffin, Exotic shorthair, local cat, and Domestic Longhair). Cats were aged between 2 and 16 years old (10.2 years old ±3.7) and weighted from 1.9 to 6.6 kg (mean 3.83 kg ± 0.9). All cats were tested for FIV and FeLV retrovirus status; four cats tested positive for FIV, and one cat was positive for FeLV.

### 3.1 Tumor Location

All of the cats were diagnosed with large‐cell lymphoma, and five of them were further classified as having B‐cell lymphoma based on immunocytochemistry (positive for PAX‐5 and negative for CD3). One cat with intestinal lymphoma with a plasmacytoid appearance was of T‐cell origin on immunocytochemistry based on positive CD3 and negative PAX‐5.

The most common tumor locations were gastrointestinal and nasopharyngeal lymphoma, each accounting for 13 cases (32%). Seven cats (17%) had mixed lymphoma, followed by three cats (7%) with renal lymphoma, two cats (5%) with multicentric lymphoma, and one cat (2%) each in mediastinal, liver, and larynx forms.

### 3.2 Chemotherapy Treatment

As standard practice in the author's clinic, most cats were started at the lower end of the dose, and the dose was escalated by 10% weekly if no adverse events were noticed. The median dose of vincristine was 0.55 mg/m^2^, the median dose of vinblastine was 1.3 mg/m^2^, and the median dose of cyclophosphamide was 239 mg/m^2^. Prednisolone was administered at the starting dose of 1.5–2 mg/kg once a day, tapered over 3–4 weeks. Asparaginase was administered in 21 cats at the discretion of the clinician before starting the chemotherapy protocol at a standard dose of 10 000 IU/m^2^ single subcutaneous injection. Animals were treated as per protocol in Table 1 with small variations depending on the case.

All cats were treated as a first‐line treatment with COVP. Cats that received the full protocol had 11 doses of vincristine, 10 doses of cyclophosphamide, and 6 doses of vinblastine. 27 cats finished inductions, and 19 cats finished the whole protocol. 19 cats relapsed and received the rescue protocol, with 17 cats receiving the LPP rescue protocol, where one cat received add‐on L‐asparaginase and cytarabine, one cat additionally received L‐asparaginase, cytarabine, and bleomycin, and three cats received add‐on L‐asparaginase, cytarabine, bleomycin, and asparaginase and modified DMAC. One cat received lomustine and cytarabine. The COP protocol was restarted in two cats after relapse.

**TABLE 2:** Clinical presentations and their respective responses and survival times of animals.

Clinical presentation (*n*)	Clinical improvement	Objective response	Median PFS (days)	MST (days)
Multicentric (*n* = 2)	2/2	1 CR, 1 PR	21, 49^a^	102, 142^a^
Mediastinal (*n* = 1)	1/1	1 CR	264^a^	264^a^
Gastrointestinal (*n* = 13)	12/13	7 CR, 2 PR, 1 SD, 3 PD	122	203
Nasopharyngeal (*n* = 13)	12/13	7 CR, 2 PR, 3 PD, 1 NE	Not reached	798
Kidney (*n* = 3)	2/3	1 CR, 1 PD, 1 NE	94, 147, 7^a^	194, 147, 7^a^
Liver (*n* = 1)	1/1	1 PR	92^a^	92^a^
Larynx (*n* = 1)	1/1	1 CR	812^a^	812^a^
Mixed (*n* = 7)	6/7	5 CR, 1 PR, 1 SD	Not reached	Not reached
Total (*n* = 41)	37/41	23 CR, 7 PR, 2 SD, 7 PD, 2 NE	264	412

*Note:* Response and survival time of 41 cats treated with the COVP protocol, ST (survival time), PFS (progression free survival), SD (stable disease), PR (partial response), PD (progressive disease), NE (not evaluated).

^a^ Individual data.

### 3.3 Adverse Events

The majority of the cats tolerated the COVP protocol well, with 86 out of all 102 adverse events (84%) being grade‐1 or grade‐2 (Table 3). Among hematological adverse events, grade‐1 neutrophilia (17%) and grade‐3 neutropenia (17%) were more common. Grade‐2 neutropenia was observed in four cats, and only two cats developed grade‐4 non‐febrile neutropenia without clinical signs. One cat with neutropenia was FIV‐positive. Seventeen cats (42%) developed anemia (grade‐1 to grade‐3). Grade‐1 vomiting was the most common COVP adverse event (24%), followed by grade‐2 reduced appetite (22%) and grade‐1 reduced appetite (20%). No grade‐3 or grade‐4 gastrointestinal adverse events were reported. None of the cats died or were hospitalized due to the treatment. Sixteen cats (39%) had a dose reduction due to adverse events; the dose of vincristine was reduced in nine cats, while the dose of vinblastine was reduced in fourteen cats (34%).

**TABLE 3:** Summary of hematological and gastrointestinal adverse events throughout the whole protocol.

	Grade‐1	Grade‐2	Grade‐3	Grade‐4
Anemia	5 (12%)	6 (15%)	6 (15%)	0 (0%)
Neutropenia	6 (15%)	4 (10%)	7 (17%)	2 (5%)
Neutrophilia	7 (17%)	3 (7%)	0 (0%)	0 (0%)
Thrombocytopenia	3 (7%)	3 (7%)	0 (0%)	0 (0%)
Thrombocytosis	1 (2%)	0 (0%)	0 (0%)	0 (0%)
Hypoglycemia	0 (0%)	0 (0%)	1 (2.4%)	0 (0%)
Decreased appetite	8 (20%)	9 (22%)	0 (0%)	0 (0%)
Vomiting	10 (24%)	5 (12%)	0 (0%)	0 (0%)
Diarrhea	4 (10%)	5 (12%)	0 (0%)	0 (0%)
Constipation	3 (7%)	0 (0%)	0 (0%)	0 (0%)
Anorexia	3 (7%)	0 (0%)	0 (0%)	0 (0%)
Melena	1 (2%)	0 (0%)	0 (0%)	0 (0%)

*Note:* Please note that this table summarizes the adverse event of the highest grade recorded in the individual cat.

The adverse events after the first dose of vincristine and vinblastine were analyzed for comparison and are listed in Table 4. The first dose of vincristine was associated with 26 hematological adverse events, whereas vinblastine was linked to 22 such events; however, grade 2–3 neutropenia events were more common with vinblastine. Furthermore, vincristine was associated with 17 gastrointestinal adverse events compared to 5 associated with vinblastine.

**TABLE 4:** Summary of hematological and gastrointestinal adverse events the week after the first administration of vincristine and vinblastine.

	Grade‐1	Grade‐2	Grade‐3	Grade‐4
Anemia	VCR: 8 VBL: 8	VCR: 6 VBL: 2	VCR: 1 VBL: 1	VCR: 1 VBL: 0
Neutropenia	VCR: 3 VBL: 1	VCR: 1 VBL: 2	VCR: 1 VBL: 2	VCR: 0 VBL: 0
Neutrophilia	VCR: 1 VBL: 6	VCR: 4 VBL: 0	VCR: 0 VBL: 0	VCR: 0 VBL: 0
Thrombocytopenia	VCR: 0 VBL: 0	VCR: 0 VBL: 0	VCR: 0 VBL: 0	VCR: 0 VBL: 0
Thrombocytosis	VCR: 0 VBL: 0	VCR: 0 VBL: 0	VCR: 0 VBL: 0	VCR: 0 VBL: 0
Hypoglycemia	VCR: 0 VBL: 0	VCR: 0 VBL: 0	VCR: 0 VBL: 0	VCR: 0 VBL: 0
Decreased appetite	VCR: 5 VBL: 3	VCR: 2 VBL: 0	VCR: 0 VBL: 0	VCR: 0 VBL: 0
Vomiting	VCR: 3 VBL: 2	VCR: 0 VBL: 0	VCR: 0 VBL: 0	VCR: 0 VBL: 0
Diarrhea	VCR: 2 VBL: 0	VCR: 0 VBL: 0	VCR: 0 VBL: 0	VCR: 0 VBL: 0
Constipation	VCR: 2 VBL: 0	VCR: 0 VBL: 0	VCR: 0 VBL: 0	VCR: 0 VBL: 0
Anorexia	VCR: 2 VBL: 0	VCR: 0 VBL: 0	VCR: 0 VBL: 0	VCR: 0 VBL: 0
Melena	VCR: 1 VBL: 0	VCR: 0 VBL: 0	VCR: 0 VBL: 0	VCR: 0 VBL: 0

*Note:* Please note that the number of events seen after the week of the first administration of VCR and VBL is summarized in this table.

Abbreviations: VBL, vinblastine; VCR, vincristine.

### 3.4 Response

Objective response could be evaluated in 39 cats. Two cats could not be evaluated for objective response because their owners noted a lack of clinical improvement and did not come back for rechecks. Based on the survival analysis in the 39 cats, the total response rate was 76% (CR + PR); 23 (59%) cats had a complete response, 7 had a partial response, 2 had stable disease, and 7 had progressive disease. 37/41 cases (90%) had an improvement in clinical signs.

#### 3.4.1 Progression‐Free Survival, Disease‐Free Interval, and Median Survival Time

**FIGURE 1:**
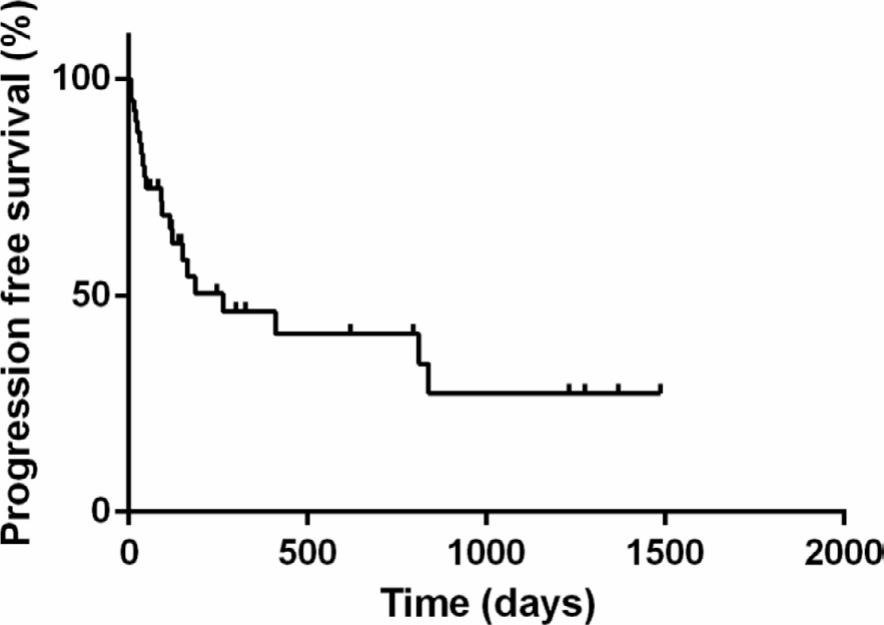
Kaplan–Meier survival curve showing overall progression‐free survival of cats treated with COVP. PFS was 264 days. Vertical bars represent censored cases.

**FIGURE 2:**
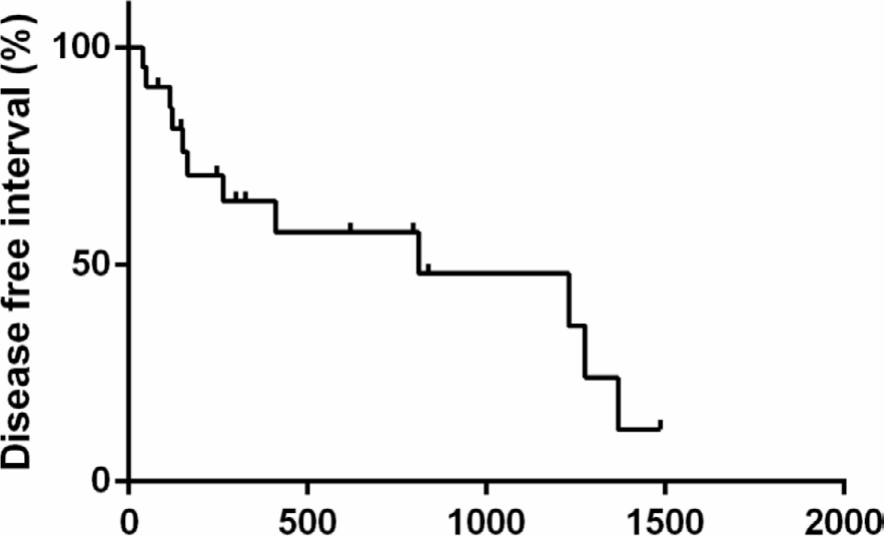
Kaplan–Meier survival curve showing the overall disease‐free interval of cats treated with COVP. DFI was 812 days. Vertical bars represent censored cases.

**FIGURE 3:**
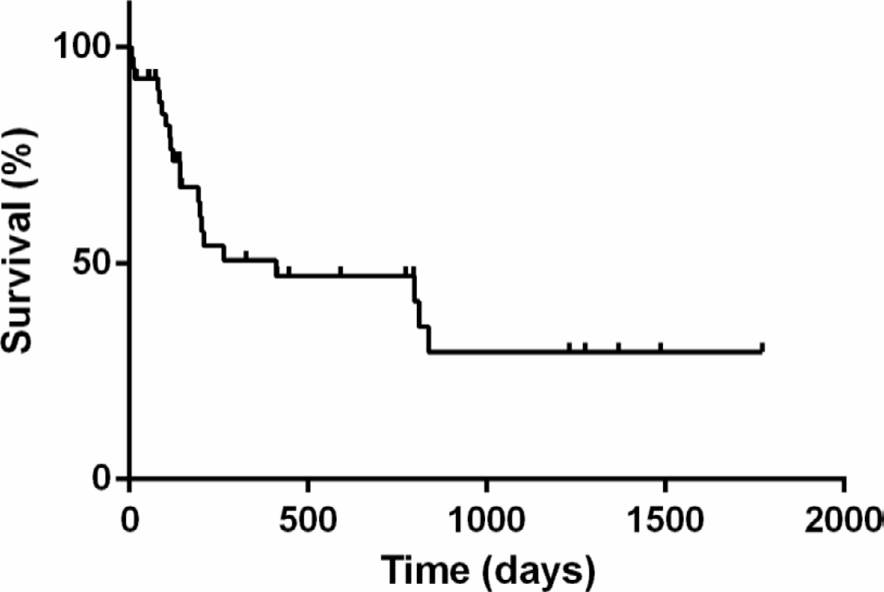
Kaplan–Meier survival curve showing the overall median survival time of cats treated with COVP. MST was 412 days. Vertical bars represent censored cases.

The PFS, DFI, and MST for animals are shown in Figures 1, 2, 3. The PFS was 264 days (range 6 to 1486 days), DFI (only for complete response) was 812 days (range 39 to 1468 days) while the MST for all cats was 412 days (range 7 to 1772 days).

**FIGURE 4:**
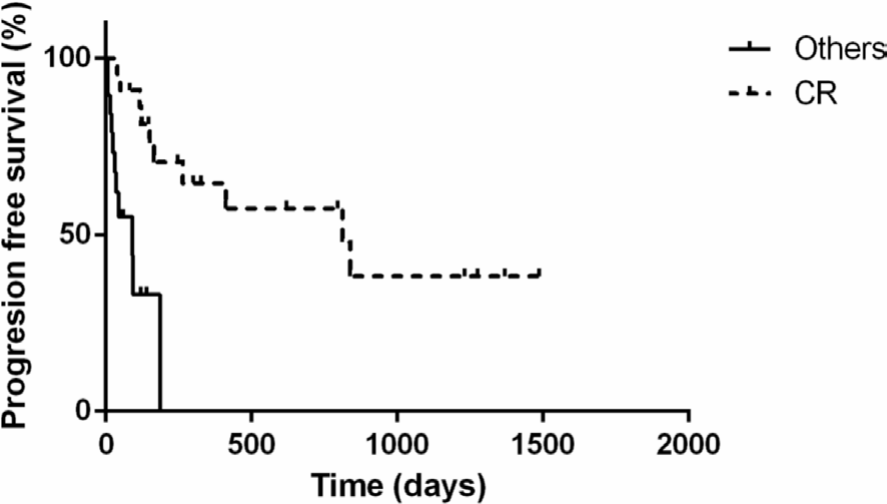
Kaplan–Meier curve depicting progression‐free survival for 41 cats with large‐cell lymphoma treated with the COVP regimen based on the initial response to COVP. Cats with complete response (PFS 812 days; dotted line) had significantly (*p* = 0.0002) longer progression‐free survival than those with partial response, stable disease, or progressive disease (PFS 92 days; solid line). Vertical bars represent censored cases.

**FIGURE 5:**
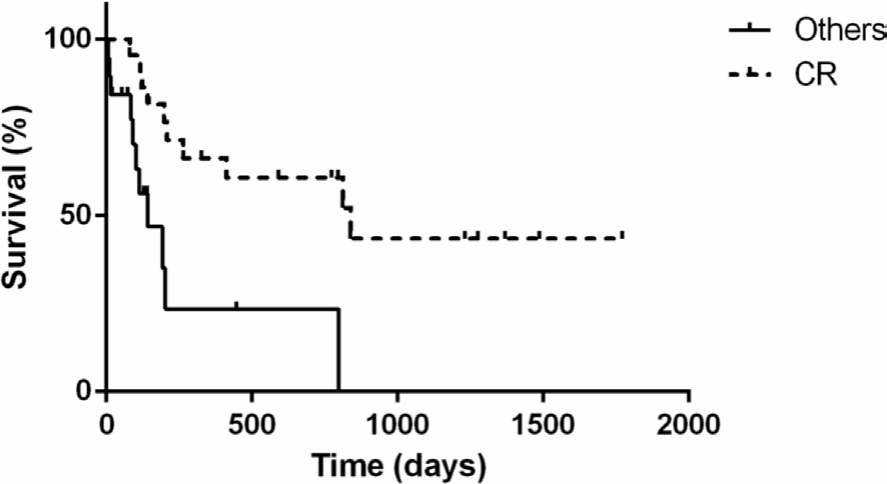
Kaplan–Meier curve depicting median survival time for 41 cats with large‐cell lymphoma treated with COVP regimen based on the initial response to COVP. Cats with complete response (MST 838 days; dotted line) had significantly (*p* = 0.0018) longer median survival time than those with partial response, stable disease, or progressive disease (MST 143 days; solid line). Vertical bars represent censored cases.

Cats that achieved complete response had significantly longer progression‐free survival (812 days; range 39 to 1486 days) compared to cats that did not achieve CR (92 days; range 6 to 187 days; *p* = 0.0002; Figure 4). Similarly, cats that achieved complete response (MST 838 days; range 81 to 1772 days) lived significantly longer (*p* = 0.0018) than those with partial response, stable disease, or progressive disease (MST 143 days; range 10 to 798 days; Figure 5).

**FIGURE 6:**
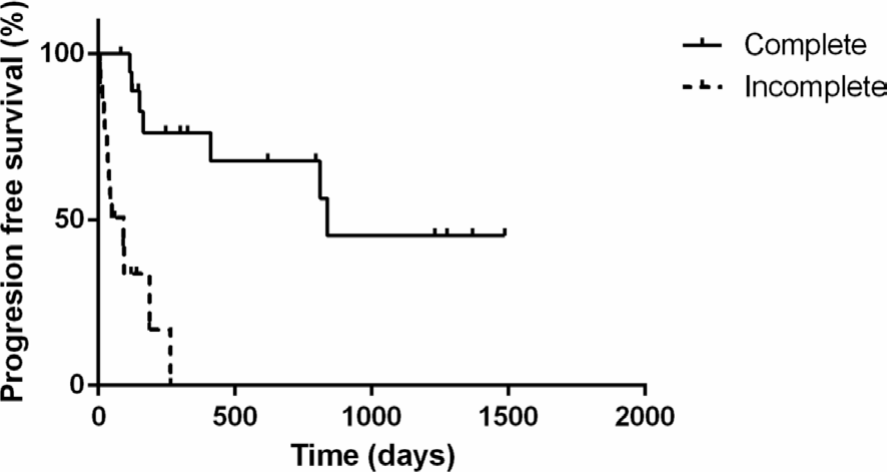
Kaplan–Meier curve depicting progression‐free survival for cats with large‐cell lymphoma treated with the COVP regimen based on the initial response to COVP. Cats that completed the treatment course had significantly longer progression‐free survival (PFS 838 days; solid line) compared to cats that did not finish treatment (PFS 92 days; dotted line; *p* < 0.0001).

Cats that completed the treatment course had significantly longer progression‐free survival (838 days; range 83 to 1486 days) compared to cats that did not finish treatment (92 days; range 6 to 264 days; *p* < 0.0001; Figure 6). Similarly, cats that had completed the treatment course (median survival not reached) lived significantly longer (*p* < 0.0001) than those who did not complete the treatment (MST 143 days; range 7 to 798 days). Most cats that did not complete treatment did not show an optimal clinical response; therefore, this is similar to what was observed in the survival curves comparing responses (Table 2).

### 3.5 Outcome

At the time of writing, 10 cats were still alive and 9 were lost at follow‐up, while 22 cats died or were euthanized. However, 3 of the 22 cats died of causes unrelated to the lymphoma. In 5 of the 22 cats that died, the cause of death was not identified and was assumed to be death from the lymphoma. The rest of the cats were either euthanized or dead due to lymphoma‐related complications. Post‐mortem examinations to examine the cause of death were not performed in any of the cases.

## 4 Discussion

The main aim of this study was to evaluate retrospectively the clinical response, survival, and safety of the COVP‐based protocol in cats with large‐cell lymphoma. This study reports a group of cats (*n* = 41) with large‐cell lymphoma treated with a new COVP protocol. Incorporating vinblastine into the COP protocol demonstrated favorable clinical efficacy compared to previous reports of the COP protocol, with adverse effects similar to or lower than those observed in previous studies involving animals receiving the COP and CHOP regimens alone [7, 19].

Vinblastine is a chemotherapy medication that is rarely used in combination protocols in cats. In humans, vinblastine is linked to reduced gastrointestinal and neurotoxic effects but a higher incidence of myelosuppression [20]. There have been a few case reports on the administration of vinblastine in cats, but no direct studies involve the use of both vincristine and vinblastine in the same chemotherapy protocol [14, 21]. Vinblastine has a similar efficacy as vincristine but with relatively fewer gastrointestinal adverse events [14]. However, the myelosuppressive effects of vinblastine are more common than with vincristine [14]. From our comparison table (Table 4), following the initial administration of vincristine and vinblastine, there was a higher incidence of grade 2–3 neutropenia adverse events and lower gastrointestinal adverse events associated with vinblastine in contrast to vincristine. This observation aligns with findings from other studies and emphasizes the potential benefits of integrating vinblastine to minimize GI adverse events [14]. In our study, we also found that neutropenia was the most adverse event noticed with vinblastine, and the target dose of 1.5 mg/m^2^ or above could not be administered.

Vincristine in cats has been considered the chemotherapy drug most frequently linked to adverse effects. In one study, the dose of vincristine was reduced in 20 out of 61 cats (33%) receiving a CHOP‐based protocol [14]. In our study, the majority of dose reductions were due to reduced appetite for vincristine and neutropenia for vinblastine. In total, 16 cats (39%) had a dose reduction, but a lower proportion of cats had a vincristine dose reduction due to the lower starting dose. It is important to highlight that the cases of neutropenia were mostly mild; none of the cases needed hospitalization, and all showed no clinical signs. This is unlikely to cause concern for the owner and unlikely to result in the owner discontinuing the chemotherapy.

Hematological adverse events were mostly mild and infrequent, with only 2 cats evidencing grade‐4 toxicoses. 42% (17/41) of cats developed anemia; however, considering that the anemia was often associated with gastrointestinal lymphoma and often improved after response to the treatment, this was probably related to the disease itself. Gastrointestinal adverse effects were more frequent, yet mostly mild and self‐resolving. It is not always possible to differentiate between gastrointestinal adverse events and clinical signs caused by gastrointestinal lymphoma; thus, gastrointestinal adverse events are also likely overestimated due to the high percentage of gastrointestinal lymphoma.

In our study, the COVP achieved an acceptable 76% response rate, with 59% of cats achieving a complete response and 17% a partial response. From extensive published literature, we showed comparable response rates, PFS, DFI, and MST. Our CR rate was 59%, which is similar to previous literature, ranging from 11% to 75% [5, 7, 14, 22, 23, 24]. The reported median survival times in various studies for the COP protocol ranged from 50 to 274 days, while our study revealed a comparably higher median survival time of 412 days [5, 7, 14, 19, 22, 23, 24]. In terms of disease‐free interval, our study demonstrated a DFI of 812 days, exceeding the previously observed range of 48 to 763 days in other studies [5, 7, 14, 19, 22, 23, 24]. Similarly, the progression‐free survival recorded in our study was 264 days, longer than the range observed in other studies (48 to 133 days) [14, 22, 24]. Therefore, these results indicate that the new protocol exhibits a possibly higher level of efficacy compared to the standard COP protocol. Cats that achieved a complete response exhibited significantly longer survival time (MST 838 days) compared to those with a partial response, stable disease, or progressive disease (MST 143 days). These findings align with similar reports from other studies [5, 7, 14, 22, 23, 24]. Anatomical types of lymphoma in cats across different publications can influence the survival outcome. Nevertheless, the measurement of COVP efficacy using MST might be less accurate compared to PFS, primarily due to factors such as cats undergoing different rescue protocols and the decision of euthanasia carried out at different stages throughout the progression of lymphoma due to the subjective nature of owners' assessment of quality of life. However, in our study, the PFS of cats that achieved CR was comparably higher than in previous studies (PFS 812 days).

The outcomes of this investigation reveal a noticeable shift to an older age distribution of cats in the present compared to two to three decades ago [25, 26]. The cats examined in this study had a median age of 10 years, which aligns with similar findings reported in recent research [25, 26].

In cats, doxorubicin usually achieves only a 30% response rate while vincristine and vinblastine achieve better response rates [8]. Vincristine and vinblastine have similar mechanisms of action, but a different mechanism of resistance and a different toxicity profile, suggesting that they could have additive or synergistic effects [14]. The main toxicity of vincristine at high dosage (0.7 mg/m^2^) is gastrointestinal, with severe constipation and megacolon also possible, while this toxicity seems less common at lower dosages (0.5 mg/m^2^) [14]. The gastrointestinal adverse events are usually the most concerning for the owner, while neutropenia without clinical signs goes unnoticed. Severe gastrointestinal adverse events are likely to lead to lower owner compliance and decline further treatment, while neutropenia can be easily resolved by adjusting future dosage. While reducing the dose of vincristine can be useful in decreasing adverse events, it could reduce the efficacy of the protocol. Adding vinblastine could increase efficacy while reducing significant adverse events. Removing vincristine completely will reduce the dose intensity of the protocol because combining vinblastine instead of vincristine with cyclophosphamide could cause severe neutropenia; hence, the drug combination used in this COVP protocol.

In our study, the most common lymphoma form was nasopharyngeal and gastrointestinal lymphoma. Nasopharyngeal lymphoma is known to have a better outcome compared to other forms of lymphoma; however, our result did not show significant differences in PFS or MST. At this stage, we cannot rule out that our relatively longer PFS could be related to the higher proportion of cases with nasopharyngeal lymphoma.

Several limitations should be acknowledged when interpreting the results of this study. The retrospective nature of this study introduces inherent bias. The small sample size and the moderate proportion of censored data in the survival analysis (20/41 [49%]) with a relatively significant proportion of cats (22%) lost at follow‐up reduce the strength of our findings. The limited size of the study group, especially when divided according to the anatomical form, also reduces its statistical power, making it challenging to achieve significant results and hindering the ability to conduct a reliable multivariate analysis. Additionally, the comparison of adverse events between vincristine and vinblastine by evaluating only the first administration does not allow for consideration of the cumulative effects.

## 5 Conclusion

In conclusion, this study describes the outcome of a group of cats with large‐cell lymphoma receiving the COVP protocol. The findings suggest that COVP treatment is safe and effective, offering an alternative treatment for cats with large‐cell lymphoma. This study validates the expected benefit of reducing the frequency and severity of gastrointestinal adverse events with vinblastine.

## Disclosure

Authors declare no off‐label use of antimicrobials.

## Ethics Statement

Authors declare no Institutional Animal Care and Use Committee or other approval was needed. Authors declare human ethics approval was not needed.

## Conflicts of Interest

The authors declare no conflicts of interest.

## References

[1] C. R. Dorn , D. O. Taylor , and H. H. Hibbard , “Epizootiologic Characteristics of Canine and Feline Leukemia and Lymphoma,” American Journal of Veterinary Research 28 (1967): 993–1001. 6070962

[2] B. Stutzer , K. Simon , H. Lutz , et al., “Incidence of Persistent Viraemia and Latent Feline Leukaemia Virus Infection in Cats With Lymphoma,” Journal of Feline Medicine and Surgery 13 (2011): 81–87. 21131219 10.1016/j.jfms.2010.09.015 PMC10822315

[3] A. Almendros , L. K. Chan , R. Dos Santos Horta , O. Nekouei , F. Hill , and A. Giuliano , “Description and Characterization of Different Types of Lymphoma in Cats in Hong Kong,” Animals (Basel) 14, no. 11 (2024): 1654, 10.3390/ani14111654. 38891700 PMC11171185

[4] E. D. Gouldin , C. Mullin , M. Morges , et al., “Feline Discrete High‐Grade Gastrointestinal Lymphoma Treated With Surgical Resection and Adjuvant CHOP‐Based Chemotherapy: Retrospective Study of 20 Cases,” Veterinary and Comparative Oncology 15 (2017): 328–335. 26333999 10.1111/vco.12166

[5] D. Simon , N. Eberle , L. Laacke‐Singer , and I. Nolte , “Combination Chemotherapy in Feline Lymphoma: Treatment Outcome, Tolerability, and Duration in 23 Cats,” Journal of Veterinary Internal Medicine 22 (2008): 394–400. 18312554 10.1111/j.1939-1676.2008.0057.x

[6] R. J. Milner , J. Peyton , K. Cooke , et al., “Response Rates and Survival Times for Cats With Lymphoma Treated With the University of Wisconsin‐Madison Chemotherapy Protocol: 38 Cases (1996–2003),” Journal of the American Veterinary Medical Association 227 (2005): 1118–1122. 16220673 10.2460/javma.2005.227.1118

[7] E. Teske , G. van Straten , R. van Noort , and G. R. Rutteman , “Chemotherapy With Cyclophosphamide, Vincristine, and Prednisolone (COP) in Cats With Malignant Lymphoma: New Results With an Old Protocol,” Journal of Veterinary Internal Medicine 16 (2002): 179–186. 11899035 10.1892/0891-6640(2002)016<0179:cwcvap>2.3.co;2

[8] A. S. Moore , S. M. Cotter , A. E. Frimberger , C. A. Wood , W. M. Rand , and D. A. L'Heureux , “A Comparison of Doxorubicin and COP for Maintenance of Remission in Cats With Lymphoma,” Journal of Veterinary Internal Medicine 10 (1996): 372–375. 8947869 10.1111/j.1939-1676.1996.tb02083.x

[9] C. H. Zwahlen , M. D. Lucroy , S. A. Kraegel , and B. R. Madewell , “Results of Chemotherapy for Cats With Alimentary Malignant Lymphoma: 21 Cases (1993–1997),” Journal of the American Veterinary Medical Association 213 (1998): 1144–1149. 9787382

[10] R. Malik , L. J. Gabor , S. F. Foster , B. E. McCorkell , and P. J. Canfield , “Therapy for Australian Cats With Lymphosarcoma,” Australian Veterinary Journal 79, no. 12 (2001): 808–817, 10.1111/j.1751-0813.2001.tb10923.x. 11837901

[11] F. Fabrizio , A. E. Calam , J. M. Dobson , et al., “Feline Mediastinal Lymphoma: A Retrospective Study of Signalment, Retroviral Status, Response to Chemotherapy and Prognostic Indicators,” Journal of Feline Medicine and Surgery 16 (2014): 637–644. 24366846 10.1177/1098612X13516621 PMC11164164

[12] S. A. Collette , S. D. Allstadt , E. M. Chon , et al., “Treatment of Feline Intermediate‐ to High‐Grade Lymphoma With a Modified University of Wisconsin‐Madison Protocol: 119 Cases (2004–2012),” Veterinary and Comparative Oncology 14, no. Suppl 1 (2016): 136–146. 26109275 10.1111/vco.12158 PMC5012421

[13] T. L. Gustafson , A. Villamil , B. E. Taylor , and A. Flory , “A Retrospective Study of Feline Gastric Lymphoma in 16 Chemotherapy‐Treated Cats,” Journal of the American Animal Hospital Association 50 (2014): 46–52. 24216491 10.5326/JAAHA-MS-5989

[14] E. L. Krick , R. B. Cohen , T. P. Gregor , P. C. Salah (Griessmayr) , and K. U. Sorenmo , “Prospective Clinical Trial to Compare Vincristine and Vinblastine in a COP‐Based Protocol for Lymphoma in Cats,” Journal of Veterinary Internal Medicine 27 (2013): 134–140. 23157371 10.1111/jvim.12006

[15] H. Madoc‐Jones and F. Mauro , “Interphase Action of Vinblastine and Vincristine: Differences in Their Lethal Action Through the Mitotic Cycle of Cultured Mammalian Cells,” Journal of Cellular Physiology 72, no. 3 (1968): 185–195. 5724569 10.1002/jcp.1040720306

[16] T. Takenaka , C. Mikuni , A. Miura , et al., “Alternating Combination Chemotherapy C‐MOPP (Cyclophosphamide, Vincristine, Procarbazine, Prednisone) and ABVd (Adriamycin, Bleomycin, Vinblastine, Dacarbazine) in Clinical Stage II–IV Hodgkin's Disease: A Multicenter Phase II Study (JCOG 8905),” Japanese Journal of Clinical Oncology 30, no. 3 (2000): 146–152. 10798542 10.1093/jjco/hyd036

[17] S. M. Nguyen , D. H. Thamm , D. M. Vail , and C. A. London , “Response Evaluation Criteria for Solid Tumours in Dogs (v1.0): A Veterinary Cooperative Oncology Group (VCOG) Consensus Document,” Veterinary and Comparative Oncology 13, no. 3 (2015): 176–183, 10.1111/vco.12032. 23534501

[18] A. K. LeBlanc , M. Atherton , R. T. Bentley , et al., “Veterinary Cooperative Oncology Group‐Common Terminology Criteria for Adverse Events (VCOG‐CTCAE v2) Following Investigational Therapy in Dogs and Cats,” Veterinary and Comparative Oncology 19, no. 2 (2021): 311–352, 10.1111/vco.12677. 33427378 PMC8248125

[19] H. Versteegh , M. Zandvliet , L. R. Feenstra , M. M. J. M. Zandvliet , F. E. M. M. van der Steen , and E. Teske , “Feline Lymphoma: Patient Characteristics and Response Outcome of the COP‐Protocol in Cats With Malignant Lymphoma in The Netherlands,” Animals (Basel) 13, no. 16 (2023): 2667, 10.3390/ani13162667. 37627457 PMC10451823

[20] B. Chabner and D. L. Longo , Cancer Chemotherapy and Biotherapy: Principles and Practice, 2nd ed. (Lippincott‐Raven Publishers, 1996), 824.

[21] D. L. Golden and V. C. Langston , “Uses of Vincristine and Vinblastine in Dogs and Cats,” Journal of the American Veterinary Medical Association 193 (1988): 1114–1117. 3058663

[22] F. Rogato , J. B. Tanis , B. Pons Gil , C. Pittaway , C. A. Johnston , and A. Guillén , “Clinical Characterisation and Long‐Term Survival of Paediatric and Juvenile Lymphoma in Cats: 33 Cases (2008–2022),” Journal of Small Animal Practice 64 (2023): 788–796. 37565270 10.1111/jsap.13667

[23] A. Krupa , J. de Vos , L. Van Eetvelde , et al., “Pegylated Asparaginase in Feline High‐Grade Lymphoma: Clinical Results of Single Injection and Continued Incorporation Into a Modified COP Regimen,” Journal of Feline Medicine and Surgery 24 (2022): e203–e213. 35748790 10.1177/1098612X221101533 PMC10812256

[24] A. H. Waite , K. Jackson , T. P. Gregor , and E. L. Krick , “Lymphoma in Cats Treated With a Weekly Cyclophosphamide‐, Vincristine‐, and Prednisone‐Based Protocol: 114 Cases (1998–2008),” Journal of the American Veterinary Medical Association 242, no. 8 (2013): 1104–1109, 10.2460/javma.242.8.1104. 23547674

[25] L. J. Gabor , R. Malik , and P. J. Canfield , “Clinical and Anatomical Features of Lymphosarcoma in 118 Cats,” Australian Veterinary Journal 76 (1998): 725–732. 9862061 10.1111/j.1751-0813.1998.tb12300.x

[26] H. Sato , Y. Fujino , J. Chino , et al., “Prognostic Analyses on Anatomical and Morphological Classification of Feline Lymphoma,” Journal of Veterinary Medical Science 76 (2014): 807–811. 24521793 10.1292/jvms.13-0260 PMC4108762

